# Prohibition in the Navy

**Published:** 1914-04

**Authors:** 


Prohibition in the Navy.—Credit for this *coup de grace* to alcohol must be given the surgeon general, Dr. Braisted. It was by his advice that Secretary Daniel issued the order; he alone could do it.

It is a matter of just pride that one of our profession has been placed in power by a wise President, and has had it in his power, and had the intelligence and good sense and courage to take this important step. It inaugurates a far-reaching reform, the ultimate good of which will be told on the race in unborn generations. I say "had the good sense," for there have been surgeons general of the navy before, and they just let the old abomination of a wine mess room for officers rock along year after year. President Wilson has now in his hand all trumps—men who do things—and certainly two right bowers. Dr. Braisted, in his letter to the Secretary of the Navy, said:

"It is difficult to find a satisfactory answer, especially in view of the youth of some of our officers, who may now be commissioned at twenty-two years of age and the proposed reduction of the minimum to twenty. To assume that even the moderate use of alcohol will better equip them physically for forty years of active service or mentally to meet responsibilities of the gravest import or intellectually solve problems which may involve our national existence or morally to represent this country at home or abroad is against all reason. It may be stated as a fact that except as a temporary expedient in certain cases of illness the use of alcohol is harmful and its abuse disastrous alike to the individual and to the human race. Its use in the service is based only on outworn customs and there is no authority by law or otherwise for its continuance except as contained in the naval instructions."

The order goes into effect July 1.

By the bye, the Secretary of the Navy's name is Daniel, and not Daniels. My name is Daniel, and not Daniels, yet everybody seems determined to have it otherwise. I suppose we will just have to "let it go at that."

---

THE LATEST ACHIEVEMENT in optical surgery was done at the Johns-Hopkins Hospital recently. The opaque cornea in a baby's eye was successfully replaced by the cornea from a pig's eye.

